# New discovery rarely runs smooth: an update on progranulin/TNFR interactions

**DOI:** 10.1007/s13238-015-0213-x

**Published:** 2015-09-25

**Authors:** Betty C. Wang, Helen Liu, Ankoor Talwar, Jinlong Jian

**Affiliations:** Stony Brook University School of Medicine, 101 Nicolls Road, Stony Brook, NY 11794 USA; New York University School of Medicine, 301 East 17th Street, New York, NY 10003 USA; Union College, 807 Union Street, Schenectady, NY 12308 USA

**Keywords:** progranulin, Atsttrin, TNFR, DR3, TNF-α, TL1A

## Abstract

Progranulin (PGRN) is a growth factor implicated in various pathophysiological processes, including wound healing, inflammation, tumorigenesis, and neurodegeneration. It was previously reported that PGRN binds to tumor necrosis factor receptors (TNFR) and has therapeutic effects in inflammatory arthritis (Tang et. al, in Science 332:478–484, 2011); however, Chen et al. reported their inability to demonstrate the PGRN-TNFR interactions under their own conditions (Chen et. al, in J Neurosci 33:9202–9213, 2013). A letter-to-editor was then published by the original group in response to the Chen et al. paper that discussed the reasons for the latter’s inability to recapitulate the interactions. In addition, the group published follow-up studies that further reinforced and dissected the interactions of PGRN-TNFR. Recently, the dispute about the legitimacy of PGRN-TNFR interactions appears to be finally settled with independent confirmations of these interactions in various conditions by numerous laboratories. This review presents a chronological update on the story of PGRN-TNFR interactions, highlighting the independent confirmations of these interactions in various diseases and conditions.

## INTRODUCTION

Progranulin (PGRN), also known as granulin-epithelin precursor (GEP), proepithelin (PEPI), acrogranin, and GP88/PC-cell derived growth factor (PCDGF), is a 593-amino-acid autocrine growth factor. PGRN contains seven-and-a-half repeats of a cysteine-rich motif (CX5-6CX5CCX8CCX6CCXDX2HCCPX4CX5-6C) in the order P-G-F-B-A-C-D-E where A-G are full repeats and P is a half-motif (Bateman and Bennett, 2009; Jian et al., 2013a). PGRN is among the earliest extracellular regulatory proteins still employed by multicellular animals (Palfree et al., 2015). Originally, PGRN was regarded as a growth factor that physiologically promotes cell proliferation and wound healing (He et al., 2003). Simultaneously, upregulated expression of PGRN was found in many types of cancer, and PGRN was found to promote cancer cell growth under these pathological conditions (Diaz-Cueto et al., 2012; Frampton et al., 2012; Serrero, 2003). *GRN* gene mutations were found to cause frontotemporal dementia (Baker et al., 2006; Cruts et al., 2006) and have been associated with other neurodegenerative diseases, such as Parkinson’s disease, Creutzfeldt-Jakob disease, motor neuron disease, and Alzheimer’s disease (Baker and Manuelidis, 2003; Lopez de Munain et al., 2008; Malaspina et al., 2001; Vercellino et al., 2011). PGRN is considered to be a neurotropic factor as well (Van Damme et al., 2008).

Besides its function as a growth factor and neurotropic factor, PGRN was reported to have anti-inflammatory activities in multiple inflammatory conditions (Jian et al., 2013a). PGRN inhibits LPS-mediated IL-6, TNF-α, and monocyte chemoattractant protein-1 (MCP-1) cytokine release from macrophages (Yin et al., 2010) and mitigates LPS-induced acute lung inflammation (Guo et al., 2012). PGRN, as opposed to its degraded granulin(s), blocks the TNF-α induced respiratory burst in neutrophils (Zhu et al., 2002). The balance between anti-inflammatory PGRN and pro-inflammatory degraded granulin units is also important in neuroinflammation (reviewed by Ahmed, Z, et al. (Ahmed et al., 2007*)*). PGRN inhibits immune complex-mediated (IC-mediated) neutrophil infiltration *in vivo* and reduces activation of isolated neutrophils by ICs *in vitro* (Kessenbrock et al., 2008). Although it was clear that PGRN had anti-inflammatory effects in these various diseases, it was uncertain how PGRN exerted these properties. The finding that PGRN directly binds to TNFR and blocks the binding of TNF-α to its receptors provided new insight into the molecular mechanisms underlying PGRN-mediated anti-inflammation (Tang et al., 2011). This review summarizes the story of PGRN-TNFR interactions, with a special focus on the recent confirmations of PGRN-TNFR interactions.

## DISCOVERY of PGRN-TNFR INTERACTIONS

PGRN was first found to bind to TNFR in a yeast two-hybrid system when screening for PGRN-binding proteins by using PGRN as bait (Tang et al., 2011). This unexpected interaction was further confirmed in human cells by co-immunoprecipitation (Co-IP). Direct protein-protein interactions between PGRN and TNFR were revealed in an ELISA-based solid phase binding assay. The binding affinities were measured through the surface plasmon resonance (SPR) approach by SensiQ (Oklahoma City, OK) and showed that PGRN selectively binds to both TNFR1 and TNFR2, and that it binds to TNFR2 with much higher affinity than TNF-α (Jian et al., 2013b; Tang et al., 2011). Notably, PGRN showed therapeutic effects in various TNF-mediated inflammatory arthritis models, including collagen-induced arthritis and spontaneous arthritis in the TNF-transgenic model (Liu, 2011; Liu and Bosch, 2012; Tang et al., 2011; Wei et al., 2014a).

The domains of PGRN required for binding to TNFR were then determined through a series of PGRN deletion mutants and three fragments composed of granulin F, A, C and their adjacent linkers were found to be responsible for the binding to TNFR. These three fragments most likely mimic the trimer structure of TNF when binding to TNFR (Wu and Siegel, 2011). Based on these results, Tang et al. further generated an engineered molecule called Atsttrin that only contained half Grn F, A, C, and was found to still bind to TNFR. Atsttrin was shown to have even better therapeutic effects than PGRN in treating inflammatory arthritis (Tang et al., 2011). In brief, this unexpected discovery of PGRN-TNFR interaction provided the molecular insights into the mechanism underlying PGRN-mediated anti-inflammatory activities and might present PGRN and its engineered derivative, Atsttrin, as the next-generation drug target for various kinds of inflammatory diseases (Liu, 2011; Liu and Bosch, 2012; Sfikakis and Tsokos, 2011; Wu and Siegel, 2011).

## INABILITY TO DEMONSTRATE THE INTERACTIONS OF PGRN-TNFR BY CHEN, ET AL.

**Figure 1:**
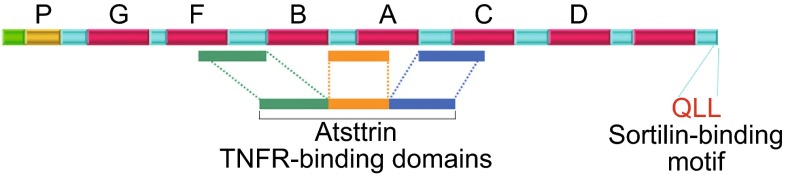
Domain structure and organization of PGRN. Three fragments required for binding to TNFR (i.e. Atsttrin) and the last three residues of PGRN (QLL) required for Sortilin binding are indicated

The findings that PGRN binds to TNFR and is therapeutic in inflammatory arthritis stimulated the explorations of PGRN/TNFR in many other inflammatory diseases and conditions (Egashira et al., 2013; Guo et al., 2012; Huang et al., 2015; Hwang et al., 2013; Kawase et al., 2013; Li et al., 2014; Liu et al., 2014; Liu et al., 2015; Thurner et al., 2015; Thurner et al., 2014; Thurner et al., 2013b; Vezina et al., 2014; Wei et al., 2014a; Yamamoto et al., 2014; Zhao et al., 2013b). However, a report by Chen et al. challenged the validity of interactions between PGRN and TNFR (Chen et al., 2013). In their paper, they claimed that they could not detect the binding of their recombinant PGRN protein to TNFR1/2 by using antibody pull-down and Biocore methods. A letter-to-editor was published by the group that originally reported PGRN-TNFR interactions, right after Chen et al. paper was released (http://www.jneurosci.org/content/33/21/9202.long/reply#jneuro_el_111445). In their response, they listed several reasons to explain Chen’s inability to demonstrate the interactions. First, the letter pointed out that the recombinant PGRN produced by Chen et al. appeared to be problematic and the data generated with their protein were inconsistent in different figures in the same paper. Second, an inappropriate chip, the CM5 chip, was used in Chen’s surface plasmon resonance assay. Proper selection of a chip is crucial for demonstrating PGRN-TNFR binding activity because some matrices encoded on the chips have been found to interfere with the interaction of PGRN with TNFR (Jian et al., 2013b). Even if the PGRN used in the Chen experiment was suitable, the PGRN-TNFR interactions would not have been observed in their SPR assay with the CM5 chip. Third, the Chen et al. paper used an improper positive control, Sortilin, to demonstrate TNFR binding activities. PGRN is a cysteine-rich protein and proper folding of the protein is critical to mediate its binding to TNFR. Successful binding to Sortilin (used as a positive control in Chen paper) is not a sufficient positive control to show PGRN-TNFR interactions because only the last three QLL amino acids of PGRN are required to bind to Sortilin (Zheng et al., 2011). The binding of PGRN to TNFR is much more complicated, and three fragments separated by other regions are needed to form a proper conformation to maintain TNFR binding activity (Fig. 1) (Tang et al., 2011).

## REINFORCEMENT OF PGRN-TNFR INTERACTIONS BY THE LABORATORY THAT ORIGINALLY REPORTED THE INTERACTIONS

**Figure 2:**
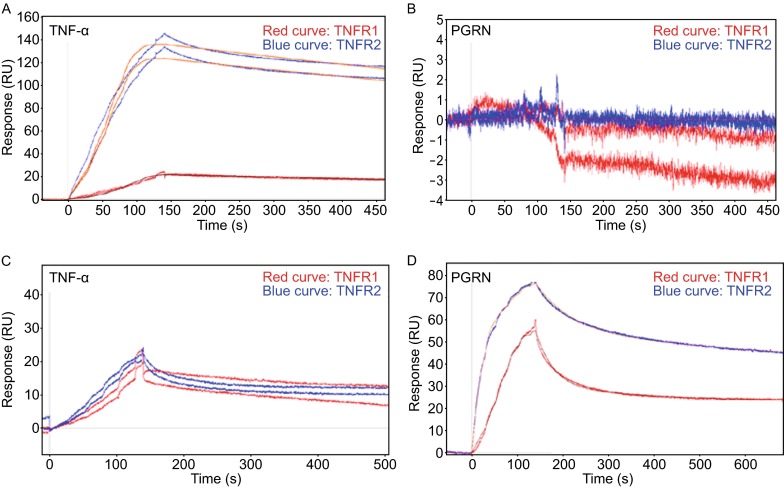
PGRN binds to TNFR on COOH1 chip, but not on COOHV chip in SensiQ surface plasmon resonance assay. (A) TNF binds to TNFR1 (red line) and TNFR2 (blue line) in SensiQ COOHV chip. (B) PGRN fails to bind to both TNFR1 (red line) and TNFR2 (blue line) on COOHV chip. (C) TNF-α binds to TNFR1α (red line) and TNFR2 (blue line) on COOH1 chip. (D) PGRN from adipogen binds to both TNFR1 (red line) and TNFR2 on COOH1 (blue line) chip. Adapted from Jian J, et al, *FEBS letters,* 2013

Surface plasmon resonance (SPR) assay is a widely used approach to demonstrate the direct interactions among macromolecules (Myszka, 1997). The type of equipment and the selection of the chip are critical in general (Maynard et al., 2009) as well as in the stoichiometry of TNF binding to TNFR (Reis et al., 2011). These proper experiment conditions also appear to be important for detecting the binding of PGRN to TNFR. The binding of PGRN to TNFR was originally detected using the COOH1 chip, a planar PEG-based chip, at SensiQ (Tang et al., 2011). This finding has now been repeated with PGRN from Adipogen (Jian et al., 2013b). However, no binding was observed using the COOHV chip, a 3D Dextran chip. In addition, the CM5 chip from Biocore (used in Chen’s paper (Chen et al., 2013)) did not detect the binding of PGRN to TNFR either. In short, the demonstration of interactions of PGRN-TNFR with SPR appears to be highly dependent upon the type of chip used. Matrix coated onto the chips, such as Dextran on COOHV chip, appears to interfere with binding of PGRN to TNFR1 and TNFR2, but does not affect the binding of TNF-α to TNFR (Fig. 2). Failure of the CM5 and COOHV chips to show PGRN-TNFR binding may be related to their high immobilization capacity, whereas a low capacity is preferable for kinetic experiments (van der Merwe et al., 1993).

**Figure 3:**
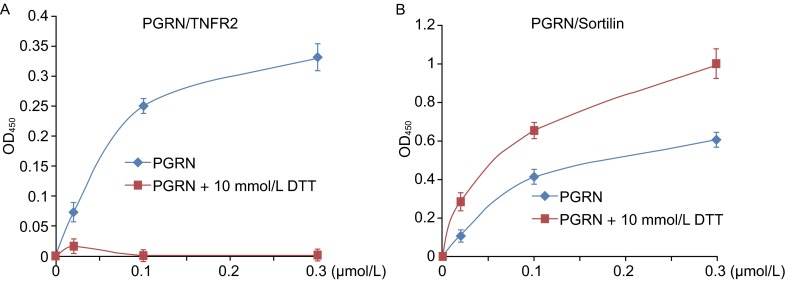
DTT treatment of PGRN abolishes its binding to TNFR, whereas enhances its binding to Sortilin. (A) DTT treatment disrupts the direct binding between PGRN and TNFR2. PGRN from Liu’s lab was pretreated with or without 10 mmol/L DTT, and various amounts of PGRN, as indicated, were coated on the plate. Binding to TNFR2 was measured by solid phase assay. (B) DTT treatment enhances the binding of PGRN to Sortilin. Similar to (A), same dosages of PGRN from Liu’s lab were pretreated with or without 10 mmol/L DTT, the binding to Sortilin was measured. Adapted from Jian J, et al, *FEBS letters,* 2013

Sortilin was set as a positive control in Chen’s paper to show that their recombinant PGRN had activity. As discussed in the follow-up paper (Jian et al., 2013b), binding to Sortilin is not sufficient to show that PGRN has proper folding, as PGRN binding to Sortilin only needs the last three QLL amino acids in the C-terminal (Zheng et al., 2011). Indeed, DTT treatment, known to disturb the conformation of PGRN, completely abolished the binding activity of PGRN to TNFR2. However, PGRN binding activity to Sortilin was actually increased after DTT pretreatment (Fig. 3) (Jian et al., 2013b), suggesting that QLL motifs at the C-terminus in the denatured PGRN might become more easily accessible to Sortilin (Fig. 1) (Jian et al., 2013b). Although addition of DTT in the *in vitro* binding system completely abolished the binding of PGRN to TNFR, it is noted that the PGRN/TNFR interactions were first detected in a yeast whose cytosol is also reducing. These results suggest that DTT may create a much stronger reducing condition, leading to the disruptions of proper disulfide bonds in PGRN, when compared to the physiological reducing conditions inside yeast cells. Additionally, some co-factors, such as chaperones known to bind to PGRN (Almeida et al., 2011), may mitigate the negative effects of reduced conditions on PGRN/TNFR interactions in yeast.

Atsttrin, consisting of half Grn F-A-C domain, also binds to TNFR and had better therapeutic effect than PGRN in inflammatory arthritis (Tang et al., 2011). It is speculated that the three fragments in Atsttrin form a conformation that mimics the trimer structure of TNF-α when binding to TNFR (Tian et al., 2014b). If this is the case, the change of the order in F-A-C should not affect the binding activity of Atsttrin to TNFR. Given this, the original order of F-A-C (Atsttrin α) was changed to A-C-F (Atsttrin β), or to A-F-C (Atsttrin γ) (Tian et al., 2014b). All three forms of Atsttrin were found to bind to TNFR in the yeast two-hybrid system. Interestingly, recombinant Atsttrin β had similar binding activity to TNFR as Atsttrin α in solid phase assay. Moreover, Atsttrin β also had a therapeutic effect in inflammatory arthritis models (Tian et al., 2014b).

## CONFIRMATION OF PGRN-TNFR INTERACTIONS IN VARIOUS DISEASES AND CONDITIONS BY INDEPENDENT LABORATORIES

Although several papers, including aforementioned follow-up studies to dissect the interaction between PGRN and TNFR (Jian et al., 2013b; Tian et al., 2014b) and detailed methodology (Tian et al., 2014a; Tian et al., 2012), were published from the laboratory that first reported the PGRN-TNFR interactions (Tang et al., 2011), the controversy about PGRN-TNFR interactions as presented by the Chen et al. paper (Chen et al., 2013) persisted. The debate appears to have finally been settled given the latest confirmations of the PGRN-TNFR interaction, as well as extensions of the interaction, in various diseases published by several independent laboratories (Alquezar et al., 2015; Li et al., 2014; Liu et al., 2014; Liu et al., 2015; Thurner et al., 2015; Wang et al., 2015a). These are briefly described below. In addition, the physical and functional associations between PGRN/Atsttrin and TNFR/DR3 as well as their interplays with TNF-α and TL1A are summarized in Table 1.

**Table 1:** Physical and functional associations of PGRN/Atsttrin and TNFR/DR3 as well as their interplays with TNF-α and TL1A

Physical/Functional interactions	Model/Methods	References
***In vitro*** **direct binding assays**		
PGRN and Atsttrin directly bind to TNFR1 and TNFR2	SPR, ELISA-based binding	Tang et al. (2011) Tian et al. (2014a)
CRD2 and CRD3 of TNFR directly bind to PGRN	SPR, ELISA-based binding	Jian et al. (2013b)
PGRN and Atsttrin bind to TNFR2 and DR3	ELISA-based binding	Liu et al. (2014)
CRD2 and CRD3 of TNFR2 inhibits the binding of PGRN to TNFR2	ELISA-based binding	Li et al. (2014)
Changing the order of F, A, C doesn’t affect Atsttrin binding to TNFR	ELISA-based binding	Tian et al. (2014b)
GST-fusion Atsttrin directly binds to EYFP-fused TNFR2	GST-pull down assay	Wang et al. (2015a)
***In vivo*** **interaction assays**		
PGRN associates with TNFR1 and TNFR2 in chondrocytes	Co-IP	Tang et al. (2011)
PGRN associates with TNFR2 in splenocytes	Co-IP	Jian et al. (2013b)
PGRN interacts with TNFR2 in chondrogenic ATDC5 cells	Co-IP	Li et al. (2014)
PGRN binds to TNFR and regulates WNT pathway in lymphocytes	Co-IP	Alquezar et al. (2015)
Phosphorylated PGRN at Serine 18 loses binding activity to TNFR and DR3 in lymphoblastoid cells	Co-IP	Thurner et al. (2015)
PGRN binds to TNFR1 and TNR2 in hepatocytes and adipocytes	Co-IP	Li et al. (2015)
**Functional interplays with TNF and TL1A**		
PGRN blocks the TNF-α induced respiratory burst in neutrophils	*In vitro* cell culture model	Zhu et al. (2002)
PGRN and Atsttrin block TNF-induced inflammatory arthritis	Inflammatory arthritis	Tang et al. (2011)
PGRN reduces LPS-induced lung inflammation through TNFR2	Acute lung injury	Guo et al. (2012)
PGRN promotes bone healing targeting TNF/TNFR signaling	Ectopic bone formation	Zhao et al. (2013a)
PGRN suppresses TNF-upregulated expression of ICAM-1 and VCAM-1 in endothelial cells	Atherosclerosis	Kawase et al. (2013)
PGNN protects ischemic-reperfusion brain injury, and directly blocks TNF-binding to neutrophils as well as neutrophil migrations	Ischemic-reperfusion brain injury	Egashira et al. (2013)
PGRN and Atsttrin have therapeutic effects to treat skin inflammation	Dermatitis	Zhao et al. (2013b)
PGRN and Atsttrin have protective role in DSS-induced colitis by blocking TNF and TL1A	Chemical-induced colitis	Wei et al. (2014a) Liu et al. (2014)
PGRN inhibits TNF-induced catabolic response and ameliorate osteoarthritis development	Spontaneous and surgically induced OA	Zhao et al. (2014)
Atsttrin ameliorates osteoarthritis development by blocking TNF-upregulated matrix proteases and inflammatory factors	Surgically induced OA	Xia et al. (2015)
3D printed Atsttrin scaffold promotes bone defect regeneration with TNF/TNFR signaling involvement	Post-calvarial defect surgery	Wang et al. (2015b)
PGRN induces insulin resistance through TNFR1 pathway	Insulin resistance	Li et al. (2015), Liu et al. (2015)
PGRN decreases hypoxia-induced renal injury	Ischemic-reperfusion kidney	Zhou et al. (2015)
**Patient data**		
PGRN overcomes TNF-α down-regulation of Treg suppressive function and Foxp3 expression PGRN autoantibodies, which provide an inflammatory environment, is detected in several autoimmune diseases Increased TNF-α activity in lymphocytes bearing FTLD *GRN* mutations	Patients samples	Tang et al. (2011) Thurner et al. (2013a) Thurner et al. (2013b) Thurner et al. (2014) Thurner et al. (2015) Alquezar et al. (2015)

### PGRN and its derivative, Atsttrin, directly bind to TNFR and DR3, and administration of Atsttrin effectively ameliorated inflammation in mouse colitis model

**Figure 4:**
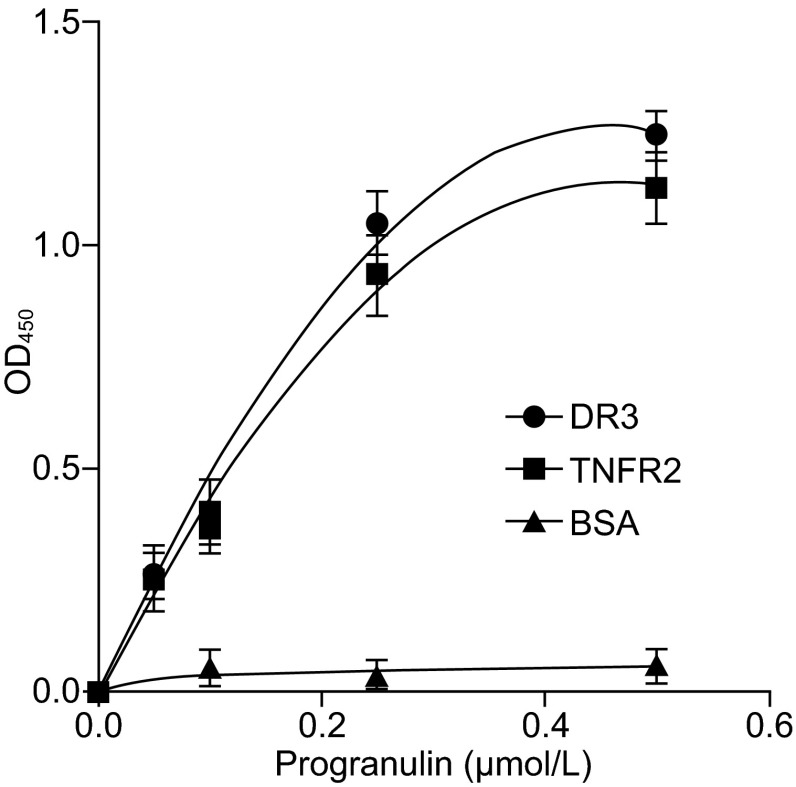
PGRN directly binds to DR3, examined by solid phase binding assay. Various dose of PGRN was coated to ELISA plate, biotinylated DR3, TNFR2 (serving as a positive control) or BSA (serving as a negative control) was then added to each well, bound protein was detected by adding avidin-HRP to each well and the absorbance was measured at OD 450 nm. Adapted from Liu C et al, *PLoS ONE* 2014

The interaction between PGRN and its derivative, Atsttrin, with TNFR was also confirmed and extended by a recent report that Atsttrin directly binds to TNFRSF25 (DR3) and inhibits TNF-like ligand 1A (TL1A) activity (Liu et al., 2014). This group screened the associations of Atsttrin with all members of the TNFR subfamily, which led to the discovery of TNFRSF25 (DR3), the highest homology to TNFR1 (Bodmer et al., 1997; Chinnaiyan et al., 1996; Croft, 2009), as an additional Atsttrin-interacting member in the TNFR family. Atsttrin inhibited the interaction between DR3 and its TNF-like ligand 1A (TL1A). In addition, Atsttrin inhibited TL1A-stimulated target gene expressions and neutralized TL1A-enhanced osteoclastogenesis *in vitro*, and ameliorated the pathology in dextran sulfate sodium induced colitis *in vivo* (Liu et al., 2014). The direct binding of Atsttrin to DR3 also led this group to examine whether or not PGRN also directly binds to DR3. Their data revealed this was the case: recombinant PGRN directly binds to DR3 in an ELISA-based *in vitro* binding assay, as it did to TNFR2 (Fig. 4).

### Direct binding between Atsttrin and TNFR2 is confirmed and quantified by a novel *in vitro* binding fluorescence assay

A novel GST pull-down-based fluorescence assay was developed by a group to detect and quantify the interaction between Atsttrin and TNFR2 (Wang et al., 2015a). In this system, GST-fusion Atsttrin was expressed in *E. coli* and purified by GST-glutathione beads. EYFP-fused TNFR2 was expressed in mammalian cells. GST-Atsttrin fusion proteins were first incubated with TNFR2-EYFP fusion proteins. After centrifugation, the complex was collected, and the interaction between Atsttrin and TNFR2-EYFP was analyzed through measuring the fluorescence intensity. The interaction between Atsttrin and TNFR2 was verified by the detection of the fluorescence after the GST-pull down. Using this modified *in vitro* approach, this group not only confirmed the direct binding of Atsttrin to TNFR, but also presented a more convenient and antibody-free assay that could be used to precisely quantify the interactions between TNFR and Atsttrin, and probably PGRN as well.

### Atsttrin in a 3D-printed scaffold promotes bone healing through targeting TNF/TNFR signaling

Recombinant PGRN protein accelerated bone formation mainly depends on TNFR2, as PGRN has a negligible effect in promoting bone formation in TNFR2 KO mice (Zhao et al., 2013a). PGRN blocked TNF-α-induced inflammatory osteoclastogenesis and protected BMP-2-mediated ectopic bone formation in TNF-α transgenic mice (Zhao et al., 2013a). Very recently, PGRN-derived Atsttrin was also reported to stimulate bone regeneration through inhibiting TNF signaling. Briefly, Atsttrin was incorporated into 3D-printed alginate (Alg)/hydroxyapatite (nHAp) scaffolds, and these scaffold implantations were used to stimulate bone regeneration (Wang et al., 2015b). Atsttrin reduced the suppressive effects of TNF-α on BMP-2-induced osteoblasts differentiation and the 3D-printed Atsttrin-Alg/nHAp scaffold significantly decreased the number of TNF-α positive cells within wound sites in a post-calvarial defect surgery model (Wang et al., 2015b).

### Intra-articular transplantation of Atsttrin-transduced mesenchymal stem cells inhibit TNF-α-mediated catabolic response and ameliorate osteoarthritis development

It was reported that aged PGRN deficient mice developed osteoarthritis (OA)-like phenotype (Zhao et al., 2014), and local injection of rPGRN into a surgically-induced OA model had therapeutic effects (Zhao et al., 2014). This protective function of PGRN in OA was independently reproduced by PGRN-derived Atsttrin recently (Xia et al., 2015). Briefly, recombinant Atsttrin is expressed in genetically-modified mesenchymal stem cells (named as MSC-Atsttrin), and MSC-Atsttrin significantly suppressed TNF-α-driven up-regulation of matrix proteases and inflammatory factors. Intra-articular injection of MSC-Atsttrin prevented the progression of degenerative changes in the surgically induced OA mouse model (Xia et al., 2015), indicating that suppression of TNF-α activity is an effective strategy for OA treatment and that intra-articular injection of MSCs-Atsttrin could be a promising therapeutic intervention.

### PGRN inhibits ER stress-mediated apoptosis primarily through interaction with TNFR2

PGRN was reported to play an important role in the ER stress response (Li et al., 2014). PGRN-deficient cells are susceptible to ER stress-induced apoptosis, and recombinant PGRN protein can rescue these cells from the ER stress-triggered signal pathway and cell death. Although PGRN was found to bind to both TNFR1 and TNFR2 in ER stress by co-immunoprecipitation (Co-IP), PGRN-mediated rescue was mainly through interaction with TNFR2 (Li et al., 2014). The CRD2 and CRD3 domains of TNFR2 are known to bind to PGRN (Jian et al., 2013b) as does to TNF-α. Recombinant GST-fusion R2C2C3 protein completely blocked PGRN binding to TNFR2 in a solid phase binding assay and further abrogated PGRN-mediated protective function in ER stress-induced cell death (Li et al., 2014). PGRN binding to TNFR2 triggered protective signaling to prevent cell death caused by ER stress. It is noted that this group also confirmed the direct interactions between PGRN and TNFR in a solid phase binding assays with recombinant proteins, in addition to the *in vivo* interactions, as assayed by Co-IP.

### PGRN binds to TNFR and regulates WNT5a expression in peripheral blood lymphocytes from FTLD-linked *GRN* mutation carriers

It is well established that *GRN* gene mutations are associated with various kinds of neurological degenerative diseases, such as frontotemporal lobar dementia (FTLD), Parkinson’s disease, Alzheimer’s disease, multiple sclerosis, and amyotrophic lateral sclerosis (Baker and Manuelidis, 2003; Lopez de Munain et al., 2008; Malaspina et al., 2001; Vercellino et al., 2011). It is also believed that inflammation plays an important role in the pathogenesis of these disorders; however, why and how PGRN deficiency associates with neuronal loss in various kinds of neurological degenerative disorders remains unknown. Very recently, Alquezar et al. reported that PGRN deficient cells showed increased expression of Wnt5a that was associated with overactivation of the NF-κB signaling (Alquezar et al., 2015). Specifically, they demonstrated the physical binding of PGRN with TNFR in lymphoblasts. The competitive nature between PGRN and TNF for binding both TNFR1 and TNFR2 was also observed. In addition, blocking TNF-activated NF-κB signaling, using either wedelolactone or specific antibodies against TNFRs, inhibited *Wnt5a* overexpression and proliferation seen in PGRN-deficient cells. In contrast, the activation of NF-κB signaling by TNF increased *Wnt5a*-dependent proliferation of control cells. Further, the overactivation of CDK6-associated kinase activity also contributed to the increase of NF-κB-mediated transcription of *Wnt5a*. Collectively, these results revealed an important role of NF-κB signaling in PGRN-associated FTLD-TDP and confirmed that PGRN binds to TNF receptors to regulate the expression of *Wnt5a*. These interesting findings not only provide new insights into the mechanism underlying *GRN* mutations associated FTLD-TDP, but also present PGRN as a potential, innovative, therapeutic target for treating patients with *GRN* mutations linked to a number of neurological degenerative diseases, especially FTLD-TDP.

### Ser81 of PGRN is critical for PGRN binding to TNFR in patients with autoimmune diseases

More interestingly, the importance of PGRN-TNFR interactions in autoimmune diseases was confirmed with patient samples in a series of studies. Progranulin autoantibody was found in patients with psoriatic arthritis (Thurner et al., 2013b) and patients positive for PGRN antibody were associated with more complications such as enthesitis or dactylitis. PGRN protective effects to TNF-induced apoptosis were inhibited by this PGRN antibody (Thurner et al., 2013b). The frequency of PGRN antibody was significantly higher in many types of autoimmune disorders, including variety of vasculitis, systemic lupus erythematosus and rheumatoid arthritis, compared with healthy control (Thurner et al., 2013a). PGRN antibody was also found in inflammatory bowel diseases as well (Thurner et al., 2014), including 16% of patients with Crohn’s disease and 21% of patients with ulcerative colitis. PGRN antibodies led to an increase of TNF-α-induced down-modulation of FOXP3 in CD4^+^CD25^hi^ Tregs (Thurner et al., 2014). The PGRN antibody was found against an epitope of hyper-phosphorylated Ser81 of PGRN (Thurner et al., 2015). PKCβ1 was identified as the relevant kinase and PP1 as the relevant phosphatase for phosphorylation and dephosphorylation of Ser81 of PGRN in patients with autoimmune diseases, and hyperphosphorylated PGRN was found to lack its binding activity to TNFR1, TNFR2 and DR3, and thereafter lost anti-TNF functions. These important findings not only confirmed the PGRN-TNFR interaction in human samples, but also indicated that certain modifications, in this case, phosphorylation at Ser81, markedly affected the interactions between PGRN and TNFR (Thurner et al., 2015).

### PGRN-TNFR1 interaction is involved in insulin resistance

The interaction between PGRN and TNFR is further ratified by investigations into PGRN’s roles in insulin sensitivity. PGRN has recently implicated in the regulation of glucose metabolism and insulin sensitivity. PGRN was found to induce impaired insulin sensitivity via its interaction with TNFR1. Notably, TNFR1 blocking peptide-Fc fusion protein was shown to block the interaction of PGRN with TNFR1 and resulted in the restoration of insulin sensitivity, showing a significant relationship between PGRN and TNFR1 (Li et al., 2015; Liu et al., 2015).

## PGRN’S ANTI-TNF ACTION IN MULTIPLE INFLAMMATORY CONTEXTS

The importance and validity of PGRN-TNFR interactions in inflammatory diseases and conditions are supported by more recent experimental and epidemiological evidences. In addition to musculoskeletal disorders, including inflammatory arthritis (Tang et al., 2011; Tian et al., 2014b), osteoarthritis (Zhao et al., 2014) and bone healing (Zhao et al., 2013a), PGRN was also reported to play an important role in other common inflammatory diseases (Table 1). For instance, PGRN is involved in the pathogenesis in the inflammatory bowel diseases. PGRN-deficient mice are susceptible to DSS and TNBS-induced colitis, whereas recombinant PGRN ameliorated the pathology and reduced the histological score. This therapeutic function is mediated through TNFR2 and IL-10, as rPGRN lost its effect in TNFR2 deficient colitis model and its therapeutic effect could be blocked by neutralizing the IL-10 antibody (Wei et al., 2014a; Wei et al., 2014b).

Expression of PGRN was significantly upregulated in response to oxazolone triggered skin inflammation (Zhao et al., 2013b). PGRN deficient mice have more severe inflammation and stronger downstream activation of the TNF signaling pathway. Atsttrin also shows its therapeutic effect for the prevention and treatment of skin inflammation (Zhao et al., 2013b). Similar results were also reported in another TPA-induced psoriasis-like skin inflammation model (Huang et al., 2015). PGRN expression was dramatically enhanced in the effected lesions of TPA-treated WT mice and PGRN deficient mice were more sensitive to the TPA-induced skin inflammation. The enhanced inflammation in PGRN KO mice is likely to be resulted from reduced regulatory T cells in the cervical lymph nodes (Huang et al., 2015). Furthermore, serum level PGRN/TNF ratio is negatively related to disease severity in psoriasis patients (Thurner et al., 2013b). PGRN-Abs are also detected with around 20% of psoriatic arthritis patients and serum levels of PGRN are significantly lower in PGRN-Abs positive patients compared with PGRN-Abs negative patient, indicating that this neutralizing PGRN Abs provides a proinflammatory environment in psoriasis patients (Thurner et al., 2013a; Thurner et al., 2013b).

In a LPS-induced acute lung injury model, intratracheal administration of LPS causes severe lung inflammation and subsequent animal death. PGRN shows remarkable reversal of LPS-induced lung permeability, as assessed by reductions in total protein, albumin, and IgM in BAL fluid (Guo et al., 2012), as well as body weight loss. This function is mediated through TNFR2, since neutralizing the antibody of TNFR2, but not TNFR1, completely blocks PGRN therapeutic function (Guo et al., 2012).

In an ischemic-reperfusion brain injury model, expression of PGRN was significantly decreased after ischemic-reperfusion injury, and supplement rPGRN protein dramatically protected neutrophil-mediated brain damage (Egashira et al., 2013), as evidenced by neutrophil infiltration, reduced levels of MMP-9, and NF-κB activation in PGRN treated group *in vivo* (Egashira et al., 2013). PGRN directly blocks TNF-binding to neutrophils as well as neutrophil migrations in a dose-dependent manner (Egashira et al., 2013).

PGRN has protective functions in atherosclerosis, as PGRN and ApoE double KO mice showed severe atherosclerotic lesions compared with ApoE KO mice. This protective function relies on PRGN anti-TNF function, as PGRN suppresses TNF-upregulated expression of ICAM-1 and VCAM-1 in endothelial cells, two crucial adhesion molecules that recruit inflammatory cells (Kawase et al., 2013). In a similar study by Hwang et al., PGRN was shown to protect vascular endothelium against atherosclerotic inflammatory reaction via Akt/eNOS and NF-kB pathway. PGRN significantly reduced the expression of TNF-α and MCP-1(Hwang et al., 2013).

PGRN is also involved in acute renal injury (Zhou et al., 2015). PGRN-deficient mice developed more severe renal damage caused by in an ischemia/reperfusion model, characterized by higher serum creatinine, increased tubular epithelial cell death, and tubulointerstitial neutrophil and macrophage infiltration when compared to WT mice (Zhou et al., 2015). Recombinant PGRN protein decreased hypoxia-induced inflammatory responses and apoptosis in proximal tubule epithelial cells *in vitro*, and further prevent or treat ischemia/reperfusion-induced renal injury *in vivo* when administered before or after ischemia (Zhou et al., 2015). Therefore, PGRN-based therapeutics might have applications in the treatment or prevention of acute kidney injury (Tadagavadi and Reeves, 2015).

## SUMMARY AND PERSPECTIVE

**Figure 5:**
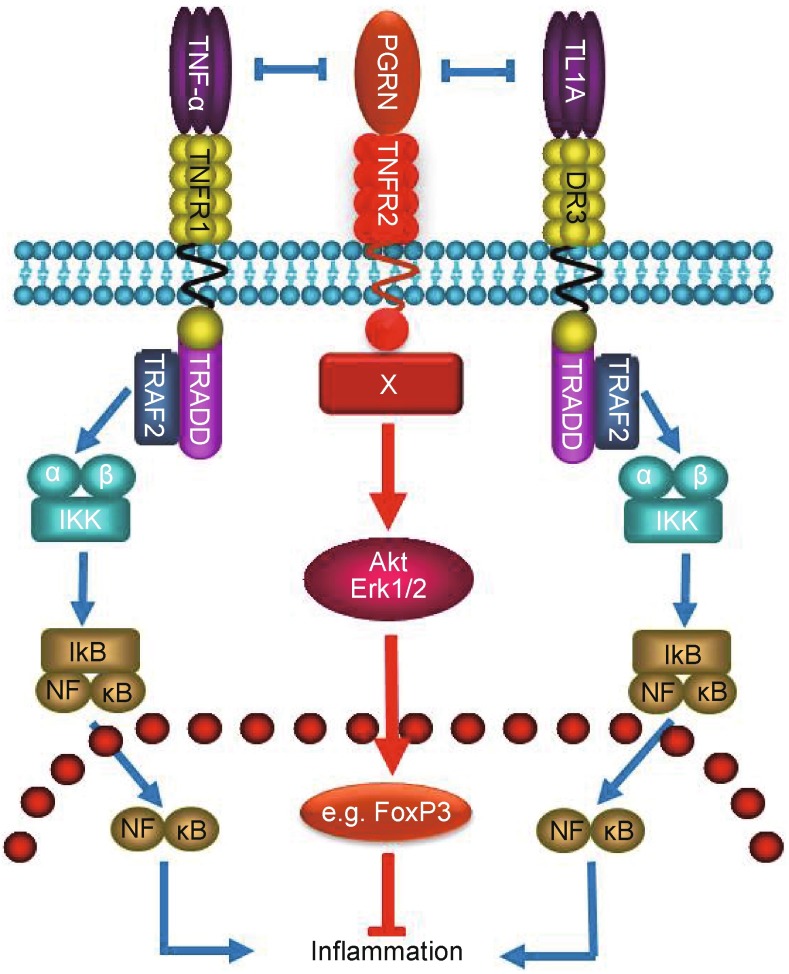
A proposed model illustrating the multiple signaling pathways by which PGRN (Atsttrin as well) exerts its anti-inflammatory actions. PGRN (Atsttrin) binds to TNFR2 and recruits unknown component, indicated by “X”, to the receptor complex(s), followed by the activation of anti-inflammatory pathway. In addition, PGRN (Atsttrin) antagonizes TNF/TNFR1 and TL1A/DR3 signaling and inhibits their inflammatory activities

PGRN is an important endogenous anti-inflammatory molecule that binds to three members in TNFR subfamily, i.e. TNFR1, TNFR2, and DR3. PGRN and its derived Atsttrin appear to exert their anti-inflammatory activities 1) by activation of PGRN/TNFR2 protective pathway through acting as a new ligand of TNFR2, which is known to mediate beneficial and protective roles in joint destruction and inflammatory processes (Aggarwal, 2014; Bluml et al., 2010; Bluml et al., 2012; Faustman and Davis, 2010; McCann et al., 2014), and 2) by inhibition of TNF/TNFR1 and TL1A/DR3 inflammatory signaling through functioning as the antagonist of TNF-α and TL1A (See proposed model in Fig. 5).

The interactions between PGRN and TNFR have been demonstrated by many laboratories in multiple disease contexts, indicating that the balance of TNF-α/PGRN is crucial to the development of these diseases. For instance, etanercept (sTNFR2) has been known to be effective in treating patients with rheumatoid arthritis (Scott, 2014), but very recent paper showed that intra-articular injection of sTNFR2 caused more severe joint destruction in a mouse model of osteoarthritis (Kimmerling et al., 2015; Olson et al., 2015). These paradoxical results may suggest the importance of PGRN-mediated protective role in the pathogenesis of osteoarthritis. This is probably because of the fact that sTNFR2 is not a TNF-α-specific inhibitor, and it is also a PGRN blocker. Actually it probably inhibits PGRN much more efficiently than does TNF-α, since PGRN exhibits an approximately 600-fold higher binding affinity to TNFR2 than TNF-α (Tang et al., 2011). TNF-α is know to be dominance in the pathogenesis of rheumatoid arthritis, and blocking TNF-α with sTNFR2 is thus beneficial to the patients with rheomaotid arthritis, whereas in the case of osteoarthritis, PGRN/TNFR2 protective/anabolic pathway is likely to outweigh TNF-α/TNFR inflammatory/catabolic signaling in regulating osteoarthritis development, thus blocking both pathways with sTNFR2 leads to more severe osteoarthritis. Interestingly, blocking TNF-α action with its specific inhibitors, such as infliximab (mouse TNF-α monoclonal antibody) and adalimumab (human TNF-α monoclonal antibody), were reported to be protective for articular cartilage and subcondral bone in animal osteoarthritis models (Ma et al., 2015; Urech et al., 2010; Zhang et al., 2012). In addition, infliximab and adalimumab were also reported to significantly alleviate signs and symptoms in patients with osteoarthritis (Grunke and Schulze-Koops, 2006; Maksymowych et al., 2012; Verbruggen et al., 2012). The opposite effects of TNF-α specific (i.e. infliximab and adalimumab) and non-specific (i.e. etanercept) inhibitors in osteoarthritis indicate the critical role of other ligand(s) of TNFR, such as PGRN, in the regulation of osteoarthritis.

The findings that PGRN is a new ligand and also an antagonist of TNFR are in accordance with other examples of ligand antagonism. One example is IL-1 receptor antagonist (IL-1RA). IL-1RA is a secreted protein that binds to IL-1R and blocks IL-1 mediated signaling pathway (Conti, 1991), IL-1RA was reported to regulate IL-1 activity in various inflammation diseases (Cavalli and Dinarello, 2015). Another example is Argos, a secreted protein that is an inhibitor of the epidermal growth factor receptor (EGFR) pathway. Interestingly, the mechanism by which Argos attenuates the EGFR pathway is by sequestration of ligand and not by direct interaction with the receptor (Klein et al., 2004; Klein et al., 2008). It appears that there exists an intricate, and universal mechanism to control critical biological and pathological activities by own naturally-occurring antagonists.

In brief, the binding to TNFR of PGRN and its-derived Atsttrin have been confirmed and extended by the group that originally reported the interactions, and also by numerous independent groups. Cumulatively, these data also solidify that PGRN, and especially Atsttrin, have potential to be the next generation anti-inflammation drug candidates/targets.

## ABBREVIATIONS

Co-IP: co-immunoprecipitation
EGFR: epidermal growth factor receptor
FTLD: frontotemporal lobar dementia
GEP: granulin-epithelin precursor
IL-1RA: IL-1 receptor antagonist
MCP-1: monocyte chemoattractant protein-1
OA: osteoarthritis
PCDGF: PC-cell derived growth factor
PEPI: proepithelin
PGRN: progranulin
SPR: surface plasmon resonance
TNFR: tumor necrosis factor receptors
